# Comparison of the Prevalence of Erectile Dysfunction Between Hypertensive and Normotensive Participants: A Case-Control Study

**DOI:** 10.7759/cureus.12061

**Published:** 2020-12-13

**Authors:** Suresh Kumar, Naresh Kumar Khurana, Sameer Lohana, Manoj Kumar Khamuani, Muhammad Khizar Memon, Sidra Memon, Syeda M Hassan, Hamza Sohail

**Affiliations:** 1 Cardiology, Bolan Medical College, Quetta, PAK; 2 Cardiology, Shalamar Medical & Dental College, Lahore, PAK; 3 Cardiology, Shalamar Hospital, Lahore, PAK; 4 Internal Medicine, Liaquat University of Medical and Health Sciences, Jamshoro, PAK; 5 Internal Medicine, Jinnah Sindh Medical University, Karachi, PAK; 6 Medicine, Jinnah Sindh Medical University, Karachi, PAK

**Keywords:** erectile dysfunction, hypertension, sexual dysfunction

## Abstract

Introduction

Hypertension is a very common risk factor for erectile dysfunction (ED). In recent time, changes in lifestyle has led to an increase in the prevalence of hypertension, which has increased the risk of ED. The purpose of this study is to assess the prevalence of ED in hypertensive patients and compare various domains of sexual activity between hypertensive and normotensive participants.

Methods

This case-control study was conducted in an outpatient department of a tertiary health care hospital in Pakistan from March 2019 to September 2019. Two hundred and twelve clinically diagnosed hypertensive patients were enrolled and were identified as case group. Control group consisted of 212 people, without any history of hypertension. Sexual function was assessed with the International Index of Erectile Function (IIEF).

Results

The prevalence of erectile dysfunction in hypertensive group was 61.79%, compared to 20.28% in normotensive group. Erectile weakness (OR = 4.32, CI 2.64-7.05), impaired morning erection (OR = 5.02, CI 2.98-8.47), complete erectile failure (OR = 2.32, CI 1.14-4.75), impaired spontaneous erection (OR = 5.45, CI 3.28-9.03), ejaculatory disturbances (OR = 5.20, CI 2.96-9.12) and reduced sexual interest (OR = 5.12, CI 3.04-8.64) were found to be significantly higher in patients with hypertension compared to normotensive participants.

Conclusion

This study has found ED to be prevalent in hypertensive patients. Identifying and acknowledging hypertension as a risk factor may help identify patients with ED and reinforce the clinician's importance of asking sexual history of hypertensive patients.

## Introduction

Prevalence of erectile dysfunction (ED) ranges from 3% to 76.5% in various part of world [1]. ED is defined as the inability to reach or maintain penile erection adequate for sexual intercourse; it has a negative impact on the sexual quality of life of men and their partners [2,3]. Prevalence of ED is expected to rise in the upcoming years due to increase in the life expectancy and aging of the population [4]. Common risk factors for ED include individual’s general health status, hypertension (HTN), diabetes mellitus (DM), metabolic syndrome, hyperlipidemia, smoking, and psychiatric/psychological disorders [2,5].

HTN, considered one of the most hazardous cardiovascular risk factors, is a frequent finding in men with ED, with 30%-50% of hypertensive; men have erectile dysfunction [5]. Multiple pathogeneses have been identified that link HTN and ED including involvement of Rho/Rho-associated coiled-coil containing protein kinase (Rho/ROCK) pathway, endothelial dysfunction, arteriolosclerosis, and side effects of antihypertensive medications [5]. According to The National Health Survey of Pakistan (NHSP), 18% of the adults in Pakistan have hypertension, with the incidence increasing to 33% in people above 45 years of age [6]. High prevalence of hypertension is due to high rate of urbanization where diets high in salts, calories, saturated fats and low in fruits and vegetables are consumed [7]. A study from 2003, compared the prevalence of ED in hypertensive men from three countries and found hypertensive men in Pakistan have much higher rate (80.8%) of ED, compared to Nigeria (57.4%) and Egypt (63.6%) [8]. Despite hypertension being very prevalent among Pakistani men, its effect on erectile function is not well studied. In this study, we will calculate the prevalence of ED and other parameters of sexual function in hypertensive men in Pakistan.

## Materials and methods

This case-control study was conducted in the outpatient department of tertiary healthcare hospital in Pakistan from March 2019 to September 2019. Two hundred and twelve known hypertensive patients were selected through consecutive convenient non-probability sampling and were identified as case group. Control group consisted of two hundred and twelve normotensive people, who visited the outpatient department as patients attendants. inclusion criteria for both case and control group were male above 40 years.

Age, gender, BMI, and smoking status were noted in a self-structured questionnaire. Sexual function was assessed with the International Index of Erectile Function (IIEF) [8]. After taking their informed consent, participants filled the questionnaire with IIEF themselves. A questionnaire in the local language was given and guidance was provided to participants when needed. The responses to IIEF questions were rated on 5-point scale. Total possible maximum score was 25; higher score indicating better sexual function (Table 1).

**Table 1:** Sexual function assessed with the International Index of Erectile Function.

International Index of Erectile Function (IIEF) Score	Severity of Erectile Dysfunction
22 to 25	No
17 to 21	Mild
12 to 16	Moderate
1 to 11	Severe

Data were analyzed using SPSS for Windows, version 22.0 (IBM Corp., Armonk, NY). Student T-test was performed to test the significance of difference between mean values. Chi-square was performed to test differences in categorical values between two or more groups. P-value of less than 0.05 meant that null hypothesis is not valid.

## Results

In our study, 36.79% participants in hypertensive men were obese and 24.53% in normotensive group were obese. The most common age group in both groups was 60 years and older. In this study, the prevalence of ED was found to be 61.79% in hypertensive group; while in normotensive group, it was 20.28%. Severe ED was found in 24.53% in hypertensive group, while it was 5.66% in normotensive group (Table 2).

**Table 2:** Comparison of characteristics between case and control group. SD: standard deviation; NS: not significant.

Variable	Hypertensive men (n=212)	Normotensive men (n=212)	P-value
Age, years, mean ± SD	55 ± 12	54 ± 12	NS
Age group			
30-39 years	12 (5.66%)	15 (7.08%)	NS
40-49 years	53 (25.0%)	49 (23.11%)	
50-59 years	52 (24.53%)	50 (23.58%)	
60 years and more	95 (44.81%)	98 (46.23%)	
Body mass Index			
Normal weight (< 25 kg/m^2^)	70 (33.02%)	100 (47.17%)	0.004
Overweight (25-30 kg/m^2^)	64 (30.19%)	60 (28.30%)	
Obese (> 30 kg/m^2^)	78 (36. 79%)	52 (24.53%)	
Smoking Status			
Never smoker	101 (47.64%)	143 (67.45%)	<0.00001
Ex-smoker	34 (16.04%)	28 (13.21%)	
Current Smoker	77 (36.32%)	41 (19.34%)	
Erectile Dysfunction			
Yes (<21)	131 (61.79%)	43 (20.28%)	<0.00001
No (22-25)	81 (38.21%)	169 (79.72%)	
Severe of Erectile dysfunction			
Severe (1-11)	52 (24.53%)	12 (5.66%)	<0.00001
Moderate (12-16)	38 (17.92%)	21 (9.91%)	
Mild (17-21)	41 (19.34%)	10 (4.72%)	
None (22-25)	81 (38.21%)	169 (79.92%)	

Comparison of prevalence of dysfunction in various domains of sexual activity between both groups as assessed by IIEF shows that prevalence of all sexual dysfunction was higher in hypertensive group compared to normotensive group. Erectile weakness (OR = 4.32, CI 2.64-7.05), impaired morning erection (OR = 5.02, CI 2.98-8.47), complete erectile failure (OR = 2.32, CI 1.14-4.75), impaired spontaneous erection (OR = 5.45, CI 3.28-9.03), ejaculatory disturbances (OR = 5.20, CI 2.96-9.12) and reduced sexual interest (OR = 5.12, CI 3.04-8.64) were significantly higher in patients with hypertension compared to normotensive participants (Table 3).

**Table 3:** Comparison of the prevalence of dysfunction in various domains of sexual activity as assessed by the International Index of Erectile Function.

Sexual dysfunction domain	Hypertensive men (n=212)	Normotensive men (n=212)	Odds Ratio (OR)	95% confidence interval	P-value
Erectile Weakness					
Present	82 (38.68%)	27 (12.74%)	4.32	2.64-7.05	<0.0001
Absent	130 (61.32%)	185 (87.26%)			
Impaired morning erection					
Present	78 (36.79%)	22 (10.38%)	5.02	2.98-8.47	<0.0001
Absent	134 (63.21%)	190 (89.62%)			
Complete erectile failure					
Present	26 (12.26%)	12 (5.66%)	2.32	1.14-4.75	<0.0001
Absent	186 (87.74%)	200 (94.34%)			
Impaired spontaneous erection					
Present	87 (41.04%)	24 (11.32%)	5.45	3.28-9.03	<0.0001
Absent	125 (58.96%)	188 (88.68%)			
Ejaculatory disturbances					
Present	69 (32.55%)	18 (8.49%)	5.20	2.96-9.12	<0.0001
Absent	143 (67.45%)	194 (91.51%)			
Reduced Sexual interest					
Present	79 (37.26%)	22 (10.38%)	5.12	3.04-8.64	<0.0001
Absent	133 (62.74%)	190 (89.62%)			

## Discussion

Study of ED pathophysiology, its risk factors, and treatment are important to overcome the lack of self-esteem, better relationship with partner and overall health of male patient [9-11]. In this study we analyzed the relationship of hypertension with ED and hypertension and assessed the overall sexual performance of 212 hypertensive Pakistani men, aged 30 and above, using IIEF, by comparing it normotensive participants.

In present study, ED was found in 61.79% patients with hypertension versus 20.28% in normotensive (p < 0.0001). The result is comparable with another study, where Burchardt et al. found out 68.3% of hypertensive male patients has some degree of ED. Assessing via IIEF, Burchardt et al., observed that hypertensive patients reported high severity of ED (45.2% in hypertensives versus 10% in a general population) [12]. We also encountered a similar observation in our study in which 24.53% hypertensive patients reported severe ED compared to 5.66% in normotensive group. Giuliano and his colleagues reported the same pattern where they found out that out of 67% hypertensive patients that reported ED, 80% patients classified the symptoms as bothersome [13]. The results from all these studies suggest a higher prevalence of ED in hypertensive patient with more severe symptoms reported of ED among hypertensive patients than normotensive population.

Another way to look at it is the prevalence rate of HTN in ED patients. In this regard, Sun et al. reported that 41% of ED had HTN versus 19% in age-matched men without ED [14]. Another study examined 272,325 patients with ED and observed that 42% of the patients had HTN. Moreover, this study also demonstrated that 42% of these men had hyperlipidemia, 20% had DM and 11% had depression. These studies suggest that there may be a causal relationship between CVD and ED [15].

The physiology behind erection consists of nitric oxide (NO) production that activates via cGMP pathway resulting in smooth muscle relaxation and dilation of arteries and arterioles, which expands the sinusoids of corpus cavernosa by filling them with blood [16]. The mechanism by which HTN results in ED can be understood is by the fact that long-standing HTN results in endothelial dysfunction. Endothelial dysfunction impairs the ability of arteries and arterioles to dilate properly and weaken the interaction of NO with smooth muscle causing a decrease in relaxation [17,18]. Another contributing factor of ED can be the antihypertensive drugs [18]. Several studies have shown that antihypertensive drugs such as thiazide diuretics and beta-blockers are well known in the development of ED. However, other anti-hypertensive therapies such as calcium channel blocker, alpha-blockers, and angiotensin-converting enzyme (ACE) inhibitors report little to no effect on sexual function [18-20]. Current guidelines recommend exploring symptoms of ED before initiating antihypertensive, assessing the symptoms during the treatment, stopping and substituting the offending drug with another antihypertensive when the symptoms of ED manifests, intervening with phosphodiesterase-5 (PDE-5) inhibitors, and avoid concomitant use of nitrates and PDE-5 inhibitors in the patient due to fear of severe hypotension [21].

ED is very prevalent in hypertensive men in Pakistan. It is an important assessment about sexual function that should be made a routine part of management of hypertensive patients. Patients should be counselled about ED as a complication of hypertension and adverse event of various anti-hypertensive medications.

## Conclusions

In our study, we also compared the prevalence of dysfunction in various domains of sexual activity in hypertensive versus normotensive patients. All domains including erectile weakness, impaired morning erection, complete erectile failure, impaired spontaneous erection, ejaculatory disturbances and reduced sexual interest were significantly higher in patients with HTN compared to normotensive participants.
